# Short Toxin-like Proteins Abound in Cnidaria Genomes 

**DOI:** 10.3390/toxins4111367

**Published:** 2012-11-16

**Authors:** Yitshak Tirosh, Itai Linial, Manor Askenazi, Michal Linial

**Affiliations:** 1 Department of Biological Chemistry, Silberman Institute of Life Sciences, The Hebrew University of Jerusalem, Jerusalem 91904, Israel; Email: yitshak.tirosh@mail.huji.ac.il (Y.T.); manoras@cs.huji.ac.il (M.A.); 2 The Racah Institute of Physics, The Hebrew University of Jerusalem, Jerusalem 91904, Israel; Email: itai.linial@mail.huji.ac.il

**Keywords:** hydra, nematostella, neurotoxin, protein families, disulfide bonds, antimicrobial peptide, ion channel inhibitor, ClanTox, complete proteome, comparative proteomics

## Abstract

Cnidaria is a rich phylum that includes thousands of marine species. In this study, we focused on Anthozoa and Hydrozoa that are represented by the *Nematostella vectensis* (Sea anemone) and *Hydra magnipapillata* genomes. We present a method for ranking the toxin-like candidates from complete proteomes of Cnidaria. Toxin-like functions were revealed using ClanTox, a statistical machine-learning predictor trained on ion channel inhibitors from venomous animals. Fundamental features that were emphasized in training ClanTox include cysteines and their spacing along the sequences. Among the 83,000 proteins derived from Cnidaria representatives, we found 170 candidates that fulfill the properties of toxin-like-proteins, the vast majority of which were previously unrecognized as toxins. An additional 394 short proteins exhibit characteristics of toxin-like proteins at a moderate degree of confidence. Remarkably, only 11% of the predicted toxin-like proteins were previously classified as toxins. Based on our prediction methodology and manual annotation, we inferred functions for over 400 of these proteins. Such functions include protease inhibitors, membrane pore formation, ion channel blockers and metal binding proteins. Many of the proteins belong to small families of paralogs. We conclude that the evolutionary expansion of toxin-like proteins in Cnidaria contributes to their fitness in the complex environment of the aquatic ecosystem.

## 1. Introduction

To date, most multicellular model organisms that have been studied come from Bilateria. A glimpse of our metazoan origin can nevertheless be seen from the recently sequenced genome of the choanoflagellate *Monosiga brevicollis* [1]. The genomic information from Porifera (sponges) has contributed to the reconstruction of the relative evolutionary position of Metazoa with respect to unicellular fungi [2]. It is now clear that a more recent branch along the evolution of metazoa links the Cnidaria and the Bilateria. This separation is dated to 650–750 million years ago [3,4,5] though, some inconsistency remains in the positioning of Cnidaria in the phylogenetic tree of the ctenophores, bilaterians and sponges [6].

Cnidaria is a phylum including thousands of species that live in aquatic environments. They include the following groups: (i) Anthozoa such as sea anemones, corals and sea pens; (ii) Scyphozoa such as the jellyfish; (iii) Cubozoa such as the box jellies, and (iv) Hydrozoa, such as the Hydra [7]. Cnidarians are distinguished from other phyla by their cnidocytes, which they use to capture prey. In most Cnidarians, a “nettle” has evolved for effective injection of venom into the prey. Such a device is found in jellyfish and cubozoans [8]. Cnidaria feed on a variety of organisms from plankton to large animals. The survival success of Cnidarians over millions of years is linked to the evolution of their toxins, many of which have yet to be discovered.

The goal of this study is the identification of toxin and toxin-like proteins (collectively termed TOLIPs) in Cnidaria. The two completed genomes that were included in this study are the Sea anemone *Nematostella vectensis* [9] from the Atlantic coasts and the *Hydra magnipapillata*. The Sea anemone is a model for the underlying developmental program of the body plan [10] and the Hydra is the first sequenced representative of the Hydrozoa that includes the fire corals, siphonophores and hydrocorals [11]. 

Animal toxins and other short proteins share a compact, cysteine rich scaffold. An increasing number of proteins resembling animal-toxins have been identified in non-venomous contexts. These proteins often act as natural cell modulators. They include pore forming proteins, proteases, protease inhibitors, as well as secreted proteins that resemble cell antigens and growth factors [12]. Several predictors were developed for identifying toxin related proteins from animals. However, each such predictor focuses on only one type or property such as the conotoxins family [13], peptidases [14] or cysteine-rich proteins [15]. A strong evolutionary relationship exists between animal toxins and ancestral cysteine cross-linked proteins [16,17,18]. The most striking examples are proteins from rodents and humans that resemble snake α-neurotoxins and act as modulators in brain [19] and skin [20]. 

The exponential growth rate of raw protein sequence has driven the field to acknowledge the need for automated, robust functional inference on a genomic scale. However, routinely used genome annotation tools often overlook the weak signal of short proteins. Furthermore, mass spectrometry (MS) methods only provide partial coverage of short proteins. The lack of transcriptomic evidence and the realization that many toxins (especially from marine animals) include non-classical post-translational modifications limit the knowledge of these short proteins. Consequently, EST collections, RNA-Seq and full-length cDNA remain the preferred source in seeking out novel short bioactive proteins.

We have developed a machine-learning based classifier called ClanTox (CLssifier of ANimal TOXins) for ranking protein sequences according to their toxin-like properties. The short proteins that carry toxin activity and those that share toxin-like compact structures are collectively called TOLIPs. ClanTox creates a robust characterization of proteins that exhibit features of compact proteins, many of which resemble animal toxins [21]. We have identified novel TOLIPs in the honeybee brain [21], in viruses and in rodents [22]. Recently, a TOLIP candidate expressed in the brain of the honeybee and other insects was validated as a non-coding brain specific expressed RNA [23]. 

We applied ClanTox to the entire available Cnidaria proteome and have identified hundreds of novel candidates. We then prioritized the predicted TOLIPs in view of their key biological functions. We found 564 TOLIPs among the 17,000 short proteins from Nematostella and Hydra. The top TOLIPS (159 and 30 candidates from Nematostella and Hydra, respectively) were carefully analyzed and we were able to infer functions for most of these proteins. We conclude this analysis with a discussion of the evolutionary and functional insights achieved through the expansion of TOLIP genes in Cnidaria.

## 2. Results

### 2.1. The Cnidarian Short Proteome

**Figure 1:**
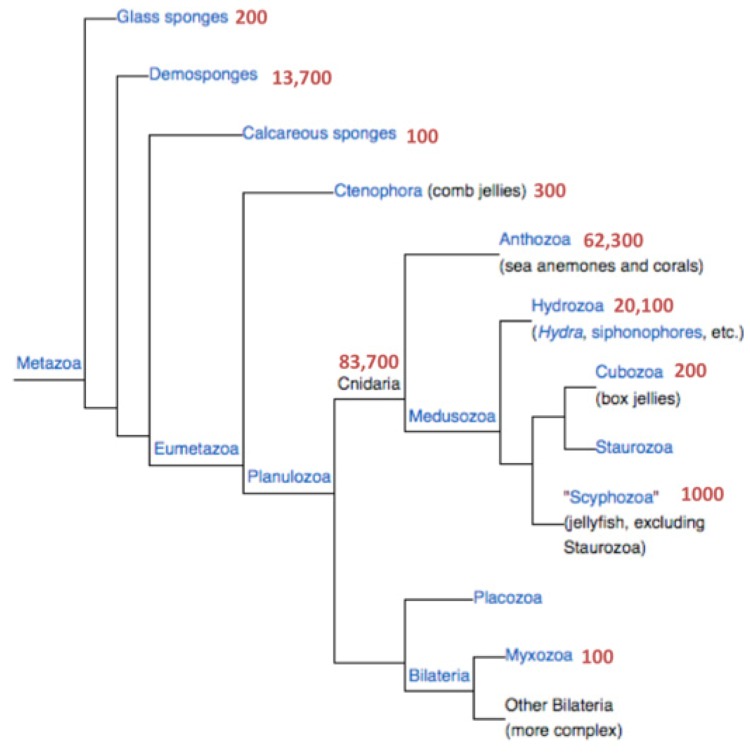
Phylogenetic tree of the metazoa. The number of protein sequences for each branch is indicated. Data are retrieved from the NCBI taxonomy database.

Currently, there are over 83,000 known proteins for the different branches of Cnidaria. Most of these sequences originate from the recently completed proteomes from the genomes of the sea anemones *Nematostella vectensis* and *Hydra magnipapillata*. Figure 1 shows the number of proteins associated with Cnidaria and the branch of the sponges. The latter are represented by *Amphimedon queenslandica* and will not be further discussed.

Short proteins are under-represented in all organisms. We have shown that a rather small number of functions populate this subset of the proteome. Notably, many of the short proteins archived in the main databases (e.g., UniProtKB [24] and NCBI Proteins) are incomplete. These databases also include fragmented sequences from incomplete mRNA sequence (*i.e.*, sequences that lack initiating Methionines or stop codons). Only a negligible percentage is attributable to processed peptides that carry a distinct biological function. The fraction of short proteins (<150 amino acids) in all eukaryotes (total 6.3 million sequences) is close to 20%. This ratio is consistent across all major branches of metazoa (e.g., Insects 19%; Echinodermata 18%). However, the proportion of short proteins in Cnidaria is significantly higher (27.5%) even relative to Porifera (Sponges, 24%). The rest of the analysis will focus exclusively on this fraction.

There are two resources for the complete proteomes from *Nematostella vectensis* and *Hydra magnipapillata* that differ in their level of redundancy. The UniProtKB reports on a total of 33,000 proteins from Cnidaria among which 9050 are short. The protein section from NCBI, on the other hand, appears to be a more complete (but somewhat redundant) resource. NCBI reports 82,400 Cnidarian sequences (Figure 1). There are 68,000 protein sequences originating from the two complete sequenced genomes of Nematostella and Hydra, 16,900 of which are short proteins (<150 amino acids, 25%). We combine these sources and focus exclusively on the short proteins (13,586 and 3314 from Nematostella and Hydra, respectively) to ensure a maximal discovery rate.

**Figure 2:**
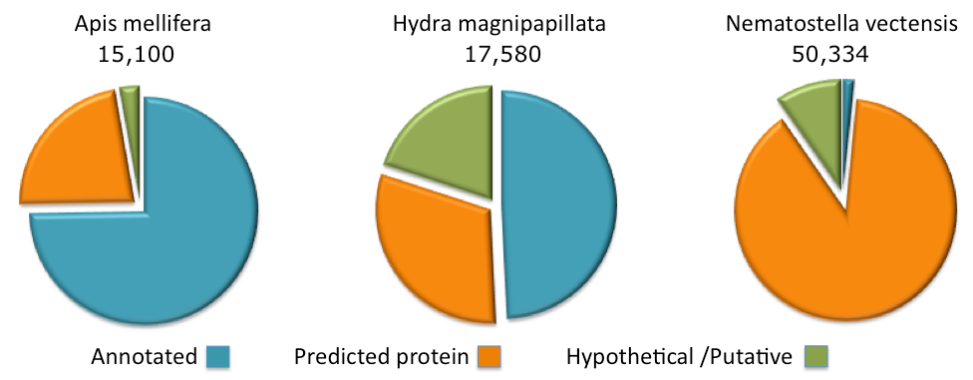
Complete proteome annotations. The fraction of the proteins annotated as predicted, hypothetical or putative are shown for *Apis mellifera*, *Hydra magnipapillata* and *Nematostella vectensis*.

### 2.2. Cnidarian Proteomes Are Partial and Poorly Annotated

The annotations of protein sequences from Cnidaria (Namatostella and Hydra) lags by comparison to the model organisms curated by the NCBI. This state of affairs is particularly extreme for the Nematostella genome where only 1.5% of the proteome has informative protein titles. The rest is indicated as “predicted” or “hypothetical” (Figure 2). For the Hydra proteome, about 50% of the sequences are associated with informative annotations and the rest are marked “predicted” or “hypothetical”. Proteomes that belong to the class of Cubozoa (sea wasps) and Scyphozoa (jellyfishes) are only partially sequenced, with less than 200 and 1000 known ORFs, respectively. 

By comparison, we present the annotation coverage of the *Apis mellifera* completed proteome. *A. mellifera* (honeybee), curation provides informative annotations for 75% of the proteome. For other species (e.g., popular model organisms), the annotation assignment of the complete proteome is higher than 75% and may reach 98% (e.g., in the case of the human proteome). Note that due to the difficulty in assigning function to short proteins, the fraction of annotated short proteins from Hydra and Nematostella is effectively negligible. 

### 2.3. Discovery of Toxin-like Proteins (TOLIPs) in Hydra

While most Hydrae are non-toxic, a few species, such as the fire coral Millepora and the Portugese Man-O-War Physalia are highly venomous animals. A bioinformatics approach for detecting bioactive peptides with toxin-like activities was conducted [25]. Surprisingly, Hydra lacks classical ion channel blockers that are found in almost all venomous organisms. However, some proteins act as Ryanodine receptor Ca^2+^ channel blockers [26]. Manual inspection reveals the complexity and richness of bioactive peptides in Hydra [25]. Among them are proteins that belong to the phospholipase family PLA2, pore forming sequences and non-classical ion channel blockers.

**Figure 3:**
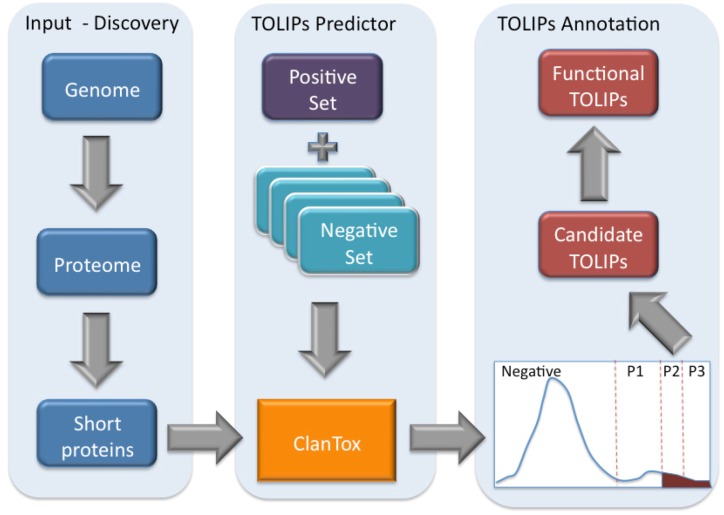
Scheme of toxin-like proteins (TOLIPs) discovery. The three major steps in TOLIPs discovery and functional inference are shown. The schematic representation of the histogram of the ClanTox prediction for short proteins is shown. The high confidence predictions are indicated as P2 and P3.

Figure 3 illustrates the schematic steps in the discovery of Cnidarian TOLIPs. The steps include the input set of short sequences, the training protocol for ClanTox, the prediction results and the semi-manual functional inference. We demonstrate the entire protocol for the Hydra short proteome whose annotation coverage is superior relative to the Nematostella proteome (Figure 2). 

ClanTox tends to identify proteins containing multiple cysteines distributed along the entire sequence. Multiple cysteines and their spacing are the hallmark of many animal secreted ion channel inhibitors. Activating ClanTox on the 17,580 proteins from the Hydra proteome revealed 110 sequences that are positively predicted to be toxin-like (marked P1–P3). 

**Figure 4:**
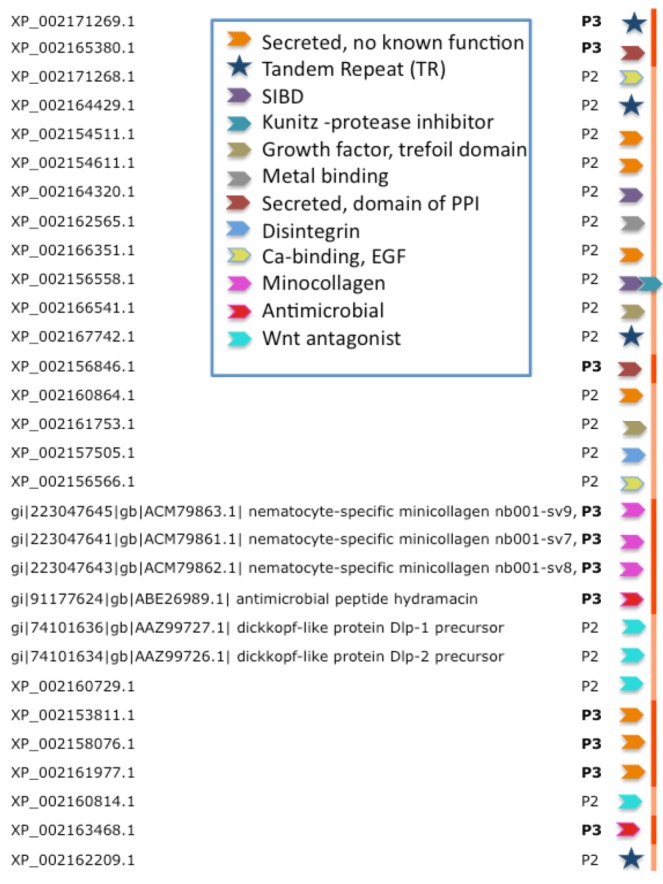
Functional inference for the predictions of TOLIPs from Hydra. TOLIPs that are listed are predicted as P2 and P3. Tandem Repeat (TR) proteins are marked by a star. All other functions are marked by colored arrowheads. XP_002156558.1 carries two functional domains.

Figure 4 lists the inferred functions for the 30 highest confidence sequences (P3 and P2, Figure 3) from Hydra. We note that four of the proteins are composed of tandem repeats (TRs). For such cases, ClanTox wrongly predicts these proteins as TOLIPs (Figure 3, stars). A protein that has even one (or more) cysteine in its repeated unit is prone to being mistakenly characterized as TOLIP. For the rest of the Hydra TOLIPs, evidence of their function can be exposed based on a homology search for domains and structural resemblance (Figure 4, arrowheads).

Figure 4 summarizes the 10 major functions associated with high-confidence predictions from Hydra. These functions include antimicrobial peptides, metal binding, protease inhibitors and domains that participate in protein interactions. Of special interest are TOLIPs that act in the Wnt signaling pathway. Reports on Wnt pathway in Cnidaria are in accord with our previously reported finding [27]. Ultimately, for about 2/3 of the predicted TOLIPs, some function can be inferred (based on Hidden Markov Models comparisons, see Experimental Section). Furthermore, for a subset of these findings, a mode of action for the toxic effect of the protein can be envisioned. 

A salient example in our findings is the identification as TOLIPs of short proteins (XP_002166541.1 and XP_002161753.1) that structurally resemble the porcine spasmolytic proteins (pSP). The latter appears in extracellular eukaryotic proteins that are stabilized by three disulphide bonds to form a trefoil motif [28]. Possibly, the Hydra pSP-like proteins comprise unknown receptor binding or a growth factor-like domain. 

### 2.4. Expansion of Cell Modulatory Functions among TOLIPs from Hydra

Most of the Hydra TOLIPs can be considered secreted with extracellular functions (Figure 4). For example, XP_002156558.1 (135 amino acids) is a secretory protein with two regions. The *N*-terminus resembles the single insulin-like growth factor binding domain protein (SIBD-1) and the *C*-terminus resembles a Protease inhibitor of the Kunitz superfamily. An architecture that is based on this combination of domains is found in additional toxins such as the β-bungarotoxin [29]. 

Beta-bungarotoxin is a heterodimeric neurotoxin consisting of a phospholipase subunit linked by a disulfide bond to a K^+^ channel binding subunit (belonging to the Kunitz protease inhibitor superfamily). Thus, toxicity is achieved by a phospholipase that is targeted to the presynaptic membrane by way of a paired Kunitz module [30]. In the case of the Hydra, we anticipate a mode in which the Kunitz protease inhibitor domain presents the SIBD-1 to produce an effective binding. Among the 3D-solved structures (from the PDB), The Hydra Kunitz domain is similar to that of several potent toxins: β-bungarotoxin (PDB: 1BUN_B), Huwentoxin-11 (PDB: 2JOT_A), Anntoxin from the tree frog *Hyla annectans* (PDB: 2KCR_A), the snake venom of the *Bungarus fasciatus* (PDB: 1JC6_A) and the green Mamba *Dendroaspis angusticeps* (PDB: 1DTK_A). 

**Figure 5:**
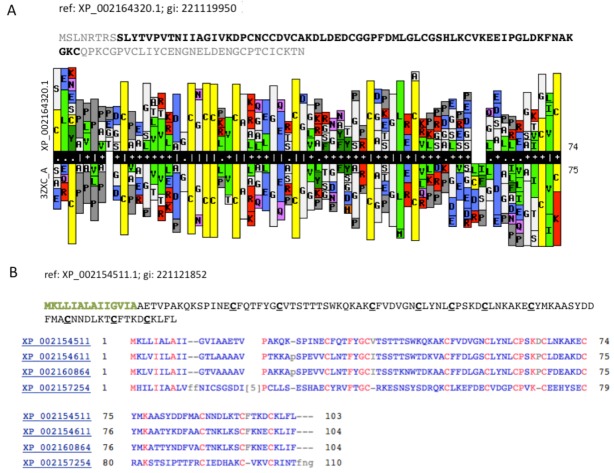
Cell modulators from Hydra. (**A**) The sequence XP_002164320.1 is shown. The segments of the sequence that were excluded from the HHPred comparative models are colored gray. HHPred representation of SIBD domain from Hydra and a structural homologue PDB: 3ZXC_A. The domain of 3ZXC includes a Single Insulin-like Growth Factor-Binding Domain Protein (SIBD-1) from the Central American Hunting Spider *Cupiennius salei*; (**B**) Set of secreted proteins and their paralogs. The function of these proteins is unknown. However, the spacing and the number of cysteines along the sequences are conserved (marked red).

We now focus on a predicted TOLIP that represents a short, secreted protein with a modulatory function. Figure 5A compares a statistical model (HMM, Hidden Markov Model) that was based on the sequence XP_002164320.1 (105 amino acids) with a library of HMM models from all 3D solved structures that are archived in the PDB. The resulting model was based on PDB accession 3ZXC from the Central America hunting spider *Cupiennius salei*. This sequence is a single insulin-like growth factor binding domain protein (SIBD-1). SIBD-1 was proposed to act in the spider’s immune system. This domain appears in 10 additional remote paralogs (the closest paralog XP_002156854.1 was scored by ClanTox at a moderate P1 confidence level). 

An expansion of TOLIP genes is a general trend among the Hydra. Figure 5B shows such instance. All paralogs of XP_002154511.1 maintain the cysteine positions (Figure 5B). While the Signal peptide segment (green font) is less conserved, the 10 cysteines in addition to a number of charged amino acids are fully conserved. It is likely that these conserved amino acids participate in the folding or binding properties of these proteins.

Modulation of adhesion through the activation of the integrin signaling pathways was identified among the proposed TOLIPs. XP_002157505.1 (and five additional paralogs) resembles the vascular apoptosis-inducing protein (VAP) from *Crotalus atrox* venom (Western diamond back rattlesnake). The similarity covers the disintegrin domain. Disintegrin is a short metalloproteinase domain that appears in viper venoms and functions as potent inhibitors of platelet aggregation and integrin-dependent cell adhesion [31].

### 2.5. The Number of Tolips Is Exceptionally High among Short Proteins from Nematostella

Table 1 summarizes the prediction results from ClanTox for Nematostella and Hydra proteomes. We divided the results according to the significance of the predictions: P3 (Very High), P2 (High) and P1 (Moderate). We also indicated the number of negatives predictions (N). It is evident that the fraction of positively identified TOLIPs in Nematostella is exceptionally high (4 fold higher relative to the Hydra). As expected, the fraction of TOLIPs among the proteins that are shorter than 100 amino acids is very high (17% for P1–P3), the fraction of TOLIPs is somewhat smaller (11%) for proteins of length 101–150. However, in both cases, the fraction appears significantly larger than the equivalent Hydra. Hydra’s TOLIPs occupy 3.1% and 3.4% of the proteins for length <100 and 101–150 amino acids, respectively. We therefore investigated the origin of TOLIPs’ expansion in the sea anemone proteome. 

**Table 1:** Results of ClanTox predictions on short proteins.

Species	Range (aa)	P3 (Very high)	P2 (High)	P1 (Moderate)	% P2–P3 predictions	Negative predictions	Total
*N. vectensis*	10–100	133	253	657	6.3	5083	6126
	101–150	26	122	704	1.9	6608	7460
	10–150	159	375	1361	3.9	11691	13586
*H. magnipapillata*	10–100	8	7	19	1.4	1038	1073
	101–150	3	12	61	0.6	2164	2241
	10–150	11	19	80	0.9	3202	3314

### 2.6. False Detection of TOLIPs Is Associated with Tandem Repeats Sequences

It has been noted that the Nematosella proteome is enriched in tandem repeats (TRs). The properties of TRs have been thoroughly studied [32]. We found that the fraction of TRs among the most highly significant TOLIPs (P3, see Experimental Section) reaches 25% of the sequences. It is substantially higher than the fraction of TR appearance in the overall proteome (16%). 

**Table 2:** Tandem Repeats (TR) proteins among the top predictions from Nematostella. Each repeat was identified in two proteins (total 40 proteins) due to redundancy.

Consensus error	Copy number	Period	Repeat
0.02	3.03	38	1
0.11	2.09	35	2
0.12	2	29	3
0.03	3.05	20	4
0.07	5.26	19	5
0.08	2.17	18	6
0.04	4.58	12	7
0.03	5.5	12	8
0.04	7	11	9
0	9.2	10	10
0.04	7.78	9	11
0.06	8.75	8	12
0.18	5.38	8	13
0.07	13.25	8	14
0.03	8.71	7	15
0.06	15.71	7	16
0.08	9.14	7	17
0.07	8.71	7	18
0.04	11.17	6	19
0.07	7.67	6	20

The properties of the repeats, the repeated unit length and the copy number of the periodicity are summarized in Table 2. There are 20 types of TR units in 40 of the top 159 TOLIP predictions (Very high, P3, Table 1).

We anticipate that these 40 TR proteins are false positives and do not play a role as Toxins or Toxin-like proteins. The TR proteome is prone to false identification of TOLIPs due to the pattern of repeats that include at least one cysteine. Importantly, the length of the repeated segment (*i.e.*, Number of TR units × Unit length) occupies most of the protein length. Many of the TR proteins lack an initiator Methionine and constitute of partial sequences with no evidence for their expression (see discussion in [32]).

### 2.7. Functional Assignment of Most TOLIPs from Nematostella

**Figure 6:**
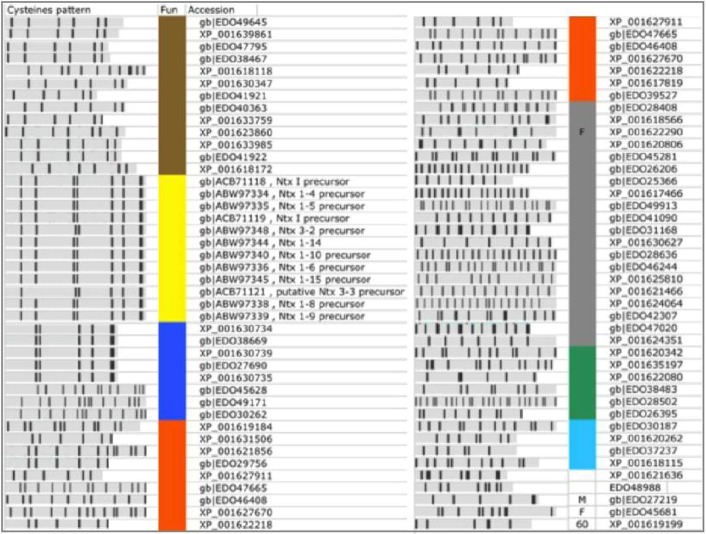
High confidence predictions from Nematostella. A list of 80 TOLIPs that were predicted by ClanTox as P3 are shown. Major functions are indicated by the colored bar next to the Cysteine pattern scheme. Neurotoxins (NTx, marked yellow) include proteins that were previously annotated as such. Predicted overlooked neurotoxins are marked blue. The other functions are colored as detailed: Gray, Tandem repeats (TR) proteins; Orange, Extracellular regulation and ligand binding; Brown, Protease inhibitors, mainly represented by the Kunitz domain; Green, homologue to specialized domains from Pfam; Light blue, Calciun modulating domains of adhesion and EGF like; M, metaloprotein; Proteins with exceptionally high number of paralogs are assigned by their number; F marks a fragment that apparently belongs to a long protein. These sequences reflect mistakes in the database assignments. The redundant list includes 159 sequences. Most proteins appear in Refseq and GeneBank and thus appear redundant by the NCBI protein database. Only the non-redundant set is shown.

Among the 119 predicted TOLIPs from Nematostella (Table 1, excluding TR proteins), 19 were already annotated as Neurotoxins. For the 100 remaining proteins, no annotations are available. These proteins are named predicted/hypothetical proteins. To assign function to these 100 proteins, we first removed the most obviously redundant proteins (*i.e.*, 100% identity in amino acids, identical length). This step led to 80 non-redundant proteins (Figure 6). Among them 20 were TR proteins (Table 2) and 12 were named Neurotoxins (Figure 6, marked yellow). 

Each sequence was tested for its most likely 3D structure using the HHpred algorithm (see Experimental Section). From this analysis we were able to annotate an additional 8 TOLIPs based on similarities to neurotoxin structural models (Figure 6, marked blue; 22 redundant proteins). These proteins can be partitioned into two main classes. The major group (5 proteins, 14 redundant proteins) shares a strong similarity to the Navs fold [33]. The Nav polypeptides (e.g., Nv1, *N. vectensis* toxin 1) inhibit the inactivation of voltage-gated sodium channels. These proteins occupy an expanded chromosomal region. Notably, changes in the expression and maturation of Nv1 transcripts are known to occur throughout the development and the life cycle of the sea anemone [33].

The other class of predicted neurotoxins is longer (range from 105–125 amino acids) with homologues from a wide array of venoms that block K^+^ channels. An example is EDO49171.1. The closest homologue of EDO49171.1 is the human EDO45628.1. The shared segment matches the MMP23 (matrix metalloproteinase 23) that is evolutionarily related to the Sea anemones peptides ShK. The ShK is a short peptide (35 amino acids) stabilized by three disulfide bridges. There are three such sequences that form a paralogous group. 

Additional functions that dominated the Nematostella’s TOLIPs belong to ligand-cell surface modulators (including Adhesion, Wnt signaling) and the Kunitz protease inhibitors (Figure 6, marked brown). These functions are shared with the Hydra TOLIPS (Figure 4).

TOLIPs that resemble adhesion domains may participate in cell-cell interaction networks. Many adhesion proteins are composed of a series of EGF-like domains that also bind calcium. For example, the protein EDO26015.1 share this domain that is found in several calcium-binding cell adhesion regulators (modeled on PDB: 2Bo2_A). Cell interaction by calcium regulation is an attractive extension of TOLIP functionality that calls for further investigation. 

In a few cases we identified TOLIPs as fragments that eventually belong to long proteins (Figure 6, F). Such cases are propagated from a failure in the genome annotation phase. From the 80 non-redundant high confidence TOLIPs, only 3% resisted functional characterization.

## 3. Discussion

The proteomes of Hydra and Nematostella are representatives of the Anthozoa and Hydrozoa that have diverged >540 million year ago [11]. These two genomes differ in their genome sizes, the GC nucleotide content, the number of transposomal elements among other features. We found that the spectrum of functions which were predicted for TOLIPs in Hydra and Nematostella proteomes overlap (compare Figure 4 and Figure 6). On the other hand, the basis for the drastic difference in the number of toxin-like candidate in each of these genomes (Table 1) is not evident. We show that 25% of the Nematostella TOLIPs are actually tandem repeat (TR) proteins. We postulate that in addition to the organism’s unique proteome, a permissive gene annotation contributes to wrongly identified sequences as TOLIP.

### 3.1. Lack of Knowledge Regarding the Cnidaria Secretome

It is expected that toxins (and TOLIPs) have a Signal peptide. However, the Cnidaria genomes are mostly un-annotated. Thus, only 11 proteins were indicated as containing a Signal peptide (SwissProt based annotation). Among the analyzed Nematostella’s predictions (P3, excluding TR proteins), we identified 34% as having a Signal peptide using SignalP 4.0. Recall that Signal peptide information was not included in the training of ClanTox. We attribute such relatively low fraction to the missing segments at the *N*-terminal of the proteins. Actually, only 32% of the analyzed short proteins from Nematostella contain an initiator Methionine. It is expected that transcriptomic data will be needed to improve the completeness of the Cnidaria sequences. 

Most Nematostella proteins (98.5%) are unannotated (Figure 2), thus the functions of their predicted TOLIPs remain elusive. Sequence search of the predicted TOLIPs highlights homologues among marine metagenomics sequences with unknown origin. For example, sequence XP_001624064.1 (130 amino acids, P3), resembles several uncharacterized sequences from metagenomic experiments. The potential for active genetic material exchange through viruses and pathogen of Cnidaria cannot be excluded. A genetic exchange from viruses to their metazoan hosts was demonstrated among short proteins [34]. However, for a number of sequences the apparent relatedness to metagenomic sequences is clearly spurious (e.g., EDO31964.1, 130 amino acids). 

### 3.2. The Cnidaria TOLIPs—A Source for New Drugs

We propose that the top Toxin-like protein predictions may lead to an expansion of known toxins, toxin-like and antibacterial proteins. Further analysis of homologs and paralogs presented in this study will lead to the identification of amino acids critical to binding and specificity. Such analysis is beyond the scope of this research. 

The therapeutic potential of TOLIPs has led to the development of toxin-based drugs [35]. Some small peptides from the conotoxin family are already in clinical use for managing chronic pain [36]. Some toxins from Nematostella act by forming pores in the targeted membranes [37]. From the mesoglea of a scyphoid jellyfish (*Aurelia aurita*) a novel antimicrobial peptide was biochemically identified with weak similarity to ion channel blockers or defensins [38]. Indeed, the Defensin-fold carries an antimicrobial activity. As such, Defensins were proposed as attractive vaccines and as potential drugs [39]. A Defensin-like fold is missing in Cnidaria. Most likely, the expansion of Defensins occurred in recently evolved phylogenetic branches prior to the speciation of Chordata. 

### 3.3. Evolution Dynamics—Expansion and Deletion of TOLIP Sequences

Representative genomes from Porifera (sponges) have provided molecular explanation for the increase in gene number due to a burst of gene duplication events. This process gave rise to the evolution of new domains (*i.e.*, adhesion molecules, lectin, proteases) [2] in Cnidaria. Our results support local expansion events of genes encoding for short proteins. The ability of a duplication burst to increase functional diversity was illustrated in yeast [40] and humans [41].

The analysis of neurotoxin (Nav1) evolution exposed extensive genomic expansion of this region [42]. Gene expansion has shaped many domain families mainly for the immune system, signaling (e.g., leucine-rich repeats) and adhesion. Several venom components evolved via convergent evolution [43]. Our study confirms that the phenomenon of genetic expansion and convergent evolution is not limited to vertebrates (e.g., reptiles, platypus) [44] but already dominates in the Cnidaria. 

## 4. Experimental Section

### 4.1. Data Collection

Protein sequences from Cnidaria were collected from UniProtKB [24] and sequences marked as “fragments” were excluded. UniProtKB was used as an annotation source for “Signal peptide” and “cell localization”. Only 1% of the Cnidaria proteins are curated and represented in the SwissProt collection (391/32,934 proteins). The proteome of *Nematostella vectensis* (Starlet sea anemone) includes 24,435 proteins in UniProtKB. The original data set was extracted from the *N. vectensis* JGI complete genome 1.0 (2007) [45]. In the case of Nematostella proteome, protein redundancy originates from accessions obtained from RefSeq and GeneBank databases. Analysis was performed on protein shorter than 150 amino acids. The FASTA file from the NCBI protein collection [46] was used as input for ClanTox prediction [47].

### 4.2. Bioinformatics Analysis Tools

SignalP 4.0 was applied for prediction of signal peptides [48]. ClustalW and alignment viewer tools were used from EBI’s (ClustalW2) server and the NCBI (Cobalt multiple sequence alignment). Multiple sequence alignment was applied using the default parameters. HHpred was used to identify remote homologues [49]. HHpred is a sensitive algorithm that is based on HMM-HMM-comparisons for proposing the most likely structure of domain family assignments. We applied HHpred to build an HMM from the query sequence and compared it with a library of HMMs representing all known 3D-structures from the PDB.

### 4.3. ClanTox Scoring

The typical performance of ClanTox as assessed by cross-validation testing is exceptionally high with a Receiver operating characteristic (ROC curve) and mean area under the curve (AUC) of >0.99% accuracy (for details see [22]). The classifier returns one of four labels: N for negative predictions and P1–P3, reflecting three levels of positive predictions for TOLIPs. The most significant set of predictions is labeled P3. The labeling P1 to P3 reflects the mean score (the higher the score, the higher is the prediction confidence), and the robustness of the score [47]. The robustness is calculated from 10 independent runs of the predictor on different negative sets and calculating the standard deviation (SD) of the prediction results. P3 comprises proteins with a mean score > 0.2. The negative predictions (*i.e.*, predicted as non-toxin) result from proteins with a mean score < −0.2. We separate the confidence of positive predictions to 3 levels: P3 are predictions with a mean score > 0.2 or mean score > 2 * SD; P2 are predictions with a mean score > 0.2 or mean score between SD and 2 * SD; P1 are predictions with a mean score > −0.2 or mean score < SD. ClanTox is accessible as an interactive web server [50].

### 4.4. Discovery of Tandem Repeats (TRs)

The presence of tandem repeats (TRs) in proteins and transcripts was determined using the Xstream web tool [51] with the following parameters: (i) TRs are >70% identical in their sequence; (ii) The minimal length of the repeated unit is 3 amino acids; (iii) The minimal domain length (defined as the total length of the repeated units) is 10 amino acids; (iv) The repeated unit appears at least twice; (v) Each repeat unit shares >80% identity to the consensus sequence; (vi) There are at most three gaps in the repeats.

## 5. Conclusions

We present here a systematic analysis for predicting Cnidarian toxin-like proteins (TOLIPs). We showed that even with poorly annotated genomes, identifying new TOLIPs candidates and inference of their possible functions is feasible. Over 95% of TOLIP candidates can be confidently annotated. For many of these predictions, experimental evidence is still lacking.

From a functional perspective, we identified candidates that are predicted to function as protease inhibitors, components of a membrane pore, ion channel blockers, metal binding proteins and signaling molecules. Importantly, many of the short compact neurotoxin folds exhibit similarity to adhesion domains (signaling and extracellular modulators). We postulate that the basic elements of adhesion in Cnidaria resemble toxin-like proteins. 

Lastly, the TOLIPs in Cnidaria belong to small families of paralogs. The identified TOLIPs from Nematostella and Hydra genomes exposed an abundance of genes that code for short templates of venom molecules. Remarkably, cysteine-rich templates account for a rich spectrum of related functions. Gene expansion dynamics is fundamental to increase the repertoire of functions with a broad range of specificity and potency. We conclude that the reported evolutionary expansion of toxin-like proteins contribute to the fitness in the complex environment of the aquatic ecosystem. 
